# Causality between serum uric acid and diabetic microvascular complications - a mendelian randomization study

**DOI:** 10.1186/s13098-024-01377-x

**Published:** 2024-06-18

**Authors:** Hongli Wu, Xuefeng Li, Wenning Zhang, Huifang Peng, Hongwei Jiang

**Affiliations:** 1 Department of Endocrinology and Metabolism, The Second Affiliated Hospital of Henan University of Science and Technology, Luoyang, China; 2 Endocrinology and Metabolism Center, The First Affiliated Hospital, and College of Clinical Medicine of Henan University of Science and Technology, Luoyang, China

**Keywords:** Serum uric acid, Diabetic microvascular complications, Mendelian randomization

## Abstract

**Background:**

The aim of this study was to investigate whether a causal relationship exists between serum uric acid (SUA) and diabetic microvascular complications using a two-sample Mendelian randomization (MR) method.

**Methods:**

We used the MR approach, utilizing genome-wide association study (GWAS) summary statistics, to estimate the causal effect of SUA on diabetic microvascular complications in European individuals. The summary statistical data of SUA were obtained from the open database (IEU OPEN GWAS PROJECT) (*p* < 5 × 10^− 8^), and data on diabetic microvascular complications (diabetic nephropathy, diabetic neuropathy, diabetic retinopathy) were obtained from the FinnGen consortium. F-statistics were calculated to assess the correlation between instrumental variables (IVs) and SUA, and single nucleotide polymorphisms (SNPs) associated with confounders or outcomes were excluded by consulting the PhenoScanner database. Inverse variance weighting (IVW) was used for primary estimation, and MR‒Egger, weighted median (WM), and Mendelian randomization pleiotropy residuals sum and outliers (MR-PRESSO) were used for additional assessment. Heterogeneity was assessed using the Cochran’s Q test, and polytropy was assessed using the MR‒Egger intercept.

**Results:**

MR analysis revealed a causal relationship between a genetically predicted increase in SUA and diabetic nephropathy [OR = 1.32, 95%(CI) = 1.07–1.63, *p* = 0.008]. The results were consistent with those after MR-PRESSO [OR = 1.30, 95%(CI) = 1.07–1.58, *p* = 0.008]. There was a causal relationship between type 2 diabetes mellitus (T2DM) and renal complication IVW [OR = 1.27, 95%(CI) = 1.00–1.62, *p* = 0.049]. These results were consistent with those after MR-PRESSO [OR = 1.27, 95%(CI) = 1.00–1.62, *p* = 0.050]. There was no significant causal relationship between the genetically predicted increase in SUA and diabetic retinopathy [OR 1.09, 95%(CI) = 0.94–1.26, *p* = 0.249] or diabetic neuropathy [OR = 1.08, 95%(CI) = 0.84–1.40, *p* = 0.549].

**Conclusions:**

This MR analysis suggests a causal relationship between genetically predicted uric acid increases and diabetic microvascular complications. A significant causal relationship exists between SUA and diabetic nephropathy but not between SUA and diabetic retinopathy or diabetic neuropathy.

**Supplementary Information:**

The online version contains supplementary material available at 10.1186/s13098-024-01377-x.

## Background

Diabetes mellitus (DM) has become one of the most widespread chronic diseases worldwide, and its morbidity continues to increase. According to International Diabetes Federation (IDF) statistics, the number of people with diabetes worldwide is expected to increase to 783.2 million in 2045 [1]. As a result of hyperglycemia and insulin resistance syndrome, the risk of macrovascular complications (e.g., cardiovascular disease) as well as microvascular complications (including diabetic nephropathy, diabetic neuropathy, and diabetic retinopathy) is significantly increased among diabetic patients. DM places a heavy financial burden on patients, their families and society, and 4.2 million adults died from DM and its complications in 2019.

Serum uric acid (SUA) is the end product of cellular purine metabolism [2] and one of the major antioxidants in plasma. SUA is capable of exerting physiological protection from antioxidants by scavenging singlet oxygen and preventing lipid peroxidation at physiological levels. Therefore, maintaining SUA within the normal range is important for regulating tissue and cellular function [3]. Many previous studies have confirmed the interaction of hyperuricemia with hypertension, DM, chronic kidney disease, coronary artery atherosclerosis, and other diseases [4]. However, some recent studies have suggested that SUA may promote the occurrence and progression of chronic diseases when it is outside the normal range. There is an association between SUA and the occurrence of diabetic microvascular complications [5, 6], but there is no uniform conclusion. Identification of this association may improve clinical monitoring and early diagnosis and prevention of diabetic microvascular complications.

Mendelian randomization (MR) is a method of causal inference based on genetic variation with the principle of random assignment of genotypes in nature and by examining the effect of genotypes on phenotypes, which makes it possible to infer the effect of biological factors on disease [7]. Specifically, MR determines mutations based on genes associated with exposure, such as single nucleotide polymorphisms (SNPs), which are used as instrumental variables (IVs) to investigate the causal relationship between exposure factors and outcome variables. The aim of this study was to investigate the causal relationship between SUA and diabetic microvascular complications, which will help clarify the role of SUA in the occurrence and progression of diabetic microvascular complications.

## Materials and methods

### Study design

**Fig. 1:**
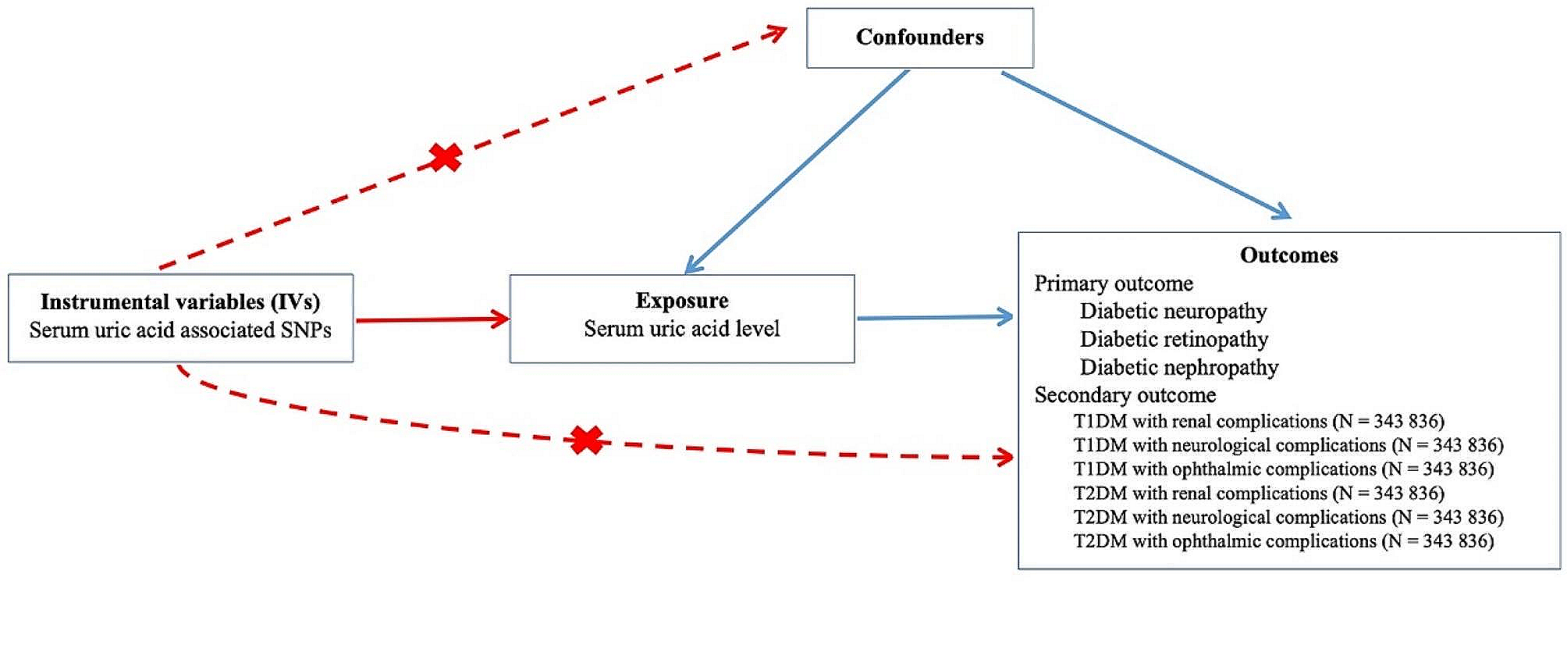
The design of two-sample Mendelian randomization. SNP: single nucleotide polymorphism; IV: instrumental variable. Solid paths indicate that IVs are associated with exposure factors, which satisfy the assumption of association; dashed paths and “x” indicate that genetic variants are not associated with confounders or cannot directly influence the outcome except through exposure pathways, satisfying the assumptions of independence and exclusivity

To investigate the causal relationship between SUA and diabetic microvascular complications, a two-sample MR analysis was designed and is shown in Fig. 1. The MR study was based on the following three hypotheses [8]: (i) the genetic variants selected as IVs are associated with SUA; (ii) the genetic variants are not associated with confounders associated with the exposure factor (uric acid) and affect the outcome factor (diabetic microvascular complications) [e.g., body mass index (BMI), blood lipids, fasting blood glucose (FBG), homocysteine, smoking, and hypertension)]; and (iii) the genetic variants influence the outcome only through the exposure factor and not through other pathways. MR findings were considered robust only if the above 3 core hypotheses were met. This study utilized recent GWAS summary statistics of SUA and diabetic microvascular complications. This article was prepared following the reporting of observational studies in the Epidemiology–Mendelian Randomization (STROBE-MR) checklist [9].

### Data sources and SNP selection

Genetic data for the exposure factor and outcome factor were obtained from public GWAS summary statistics that support statistical data downloads and systematic causal inference studies. The summary statistical data of SUA were obtained from the IEU OPEN GWAS PROJECT involving 19,041,286 SNPs from 343,836 participants of European ancestry (these data can be downloaded from https://gwasmrcieu.ac.uk/datasets, ID: ebi-a-GCST90018977). We selected GWAS summary statistics of diabetic nephropathy, diabetic retinopathy, and diabetic neuropathy from the FinnGen consortium (https://r9.finngen.fi/) [10]. The study for these patients, whose participants were all of European descent, was initiated in Finland in 2017, and cases were identified based on International Classification of Diseases codes. The details of the instrument SNPs are listed in Supplementary Table S1.

**Fig. 2:**
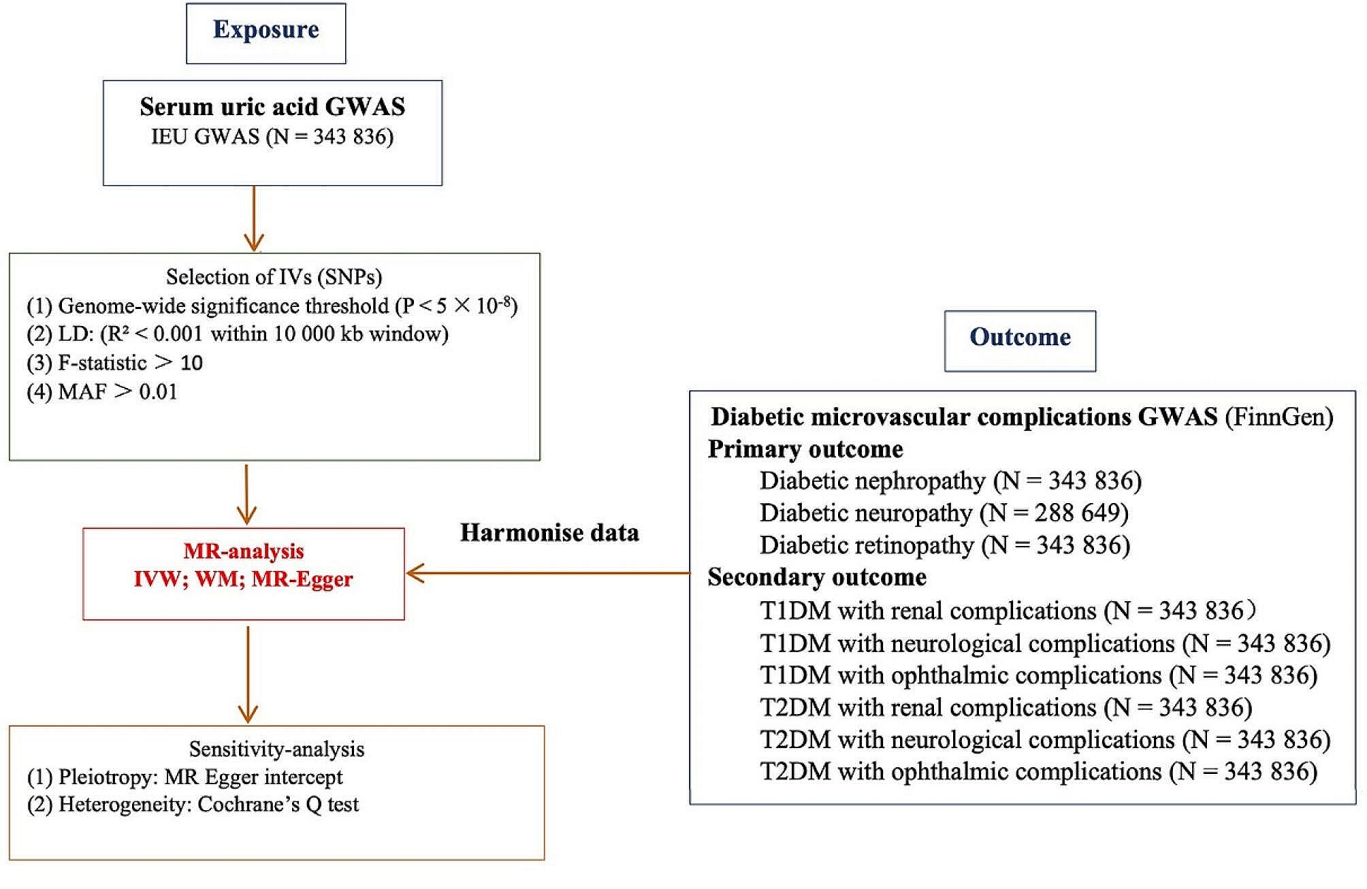
Rectangular chart of the selected instrumental variables (IVs). SNPs: single-nucleotide polymorphisms; Gwas: Genome-wide association study; LD: linkage disequilibrium; MAF: minor allele frequency; IVM: inverse variance weighted; WM: weighted median

The selection of IVs met the following requirements: (i) SNPs significantly associated with SUA were extracted from the GWAS summary statistics (*p* < 5 × 10^− 8^); (ii) to avoid bias generated by linkage disequilibrium (LD), we selected SNPs that were independent of each other as IVs; parameter conditions r^2^ = 0.001, kb = 10 000, and minor allele frequency (MAF) > 0.01 were set in R software; and (iii) we calculated F-statistics to test for weak IV bias and selected strong IVs for MR analyses where SNPs with F > 10 were defined as strong IVs and SNPs with F < 10 were defined as weak IVs. We calculated the F statistic using the formula F= (βexposure/SEexposure)^2^ (β is the effect value of the allele; SE indicates the variance of each SNP) [11]. (iv) We searched the phenoscanner database for all SNPs associated with exposure (http://www.phenoscanner.medschl.cam.ac.uk/) and removed SNPs associated with confounders or outcomes (*P* < 1 × 10^− 5^) to avoid potential pleiotropic effects. We used the screened SNPs as IVs in this study, and the rectangular chart of the selected IVs a shown in Fig. 2.

### MR analysis

The main analyses were performed using the multiplicative random effects inverse-variance weighted (IVW) method, which combines cumulative causal estimates of Wald ratios derived from each IV [8]. MR‒Egger, WM, and MR-PRESSO were used for supplementary analyses to ensure the validity and robustness of the results [12]. The results of the MR analyses are presented as odds ratios (ORs) and corresponding 95% confidence intervals (CIs). We considered the causal effect of exposure on outcome to be significant if the p value was < 0.05.

In this study, we used the horizontal pleiotropy test, heterogeneity test, and “leave-one-out” method for sensitivity analysis to ensure the reliability of the results. The MR‒Egger regression method was utilized to test the horizontal pleiotropy of selected SNPs. Its intercept test value indicates the magnitude of pleiotropy, and the closer it is to 0, the less likely it is that the gene has pleiotropy [13]. According to the results of the heterogeneity test, if the Q-statistic was greater than 0.05, it indicated no heterogeneity in the selected IVs, and the effect of heterogeneity on the causal effect could be ignored. If there was heterogeneity in the IVs, the random effects IVW method was used to estimate the causality. Otherwise, a fixed effects model was used for analysis. MR-PRESSO tests were performed to detect potential outliers and obtain corrected estimates. In addition, the “leave-one-out” method ensures the reliability of the results by assessing the influence of individual SNPs on the causal effect of exposure on outcome. The magnitude of the change in the effect size of the remaining SNPs was calculated by removing each SNP. Statistical analyses were performed using R software (version 4.0.2) with the “TwoSampleMR” (version 0.5.7) and “MR-PRESSO” (version 1.0) software packages.

## Results

### Genetic instrument selection and genetic correlation between phenotypes

F-statistics of all the IVs of SUA were above the threshold of 10, indicating that the IVs were strong instruments and thus reducing the bias of the IV estimates (Supplementary Table S2). Then, SNPs with incompatible alleles (e.g., rs2749005 and rs34555420) and palindromic SNPs whose orientation could not be determined (e.g., rs12510175, rs1851285, rs1869581, rs2252862, rs2493121, rs538737, and rs7039) were eliminated by data harmonization. Furthermore, 21 SNPs associated with the outcome variable, i.e., diabetic microvascular complications (e.g., BMI, blood lipids, FBG, homocysteine, smoking status, and hypertension status), were identified and excluded by screening in the PhenoScanner database (Supplementary Table S3). Finally, the screened SNPs were used as IVs of the SUA.

### Causal effect of SUA on diabetic nephropathy

**Fig. 3:**
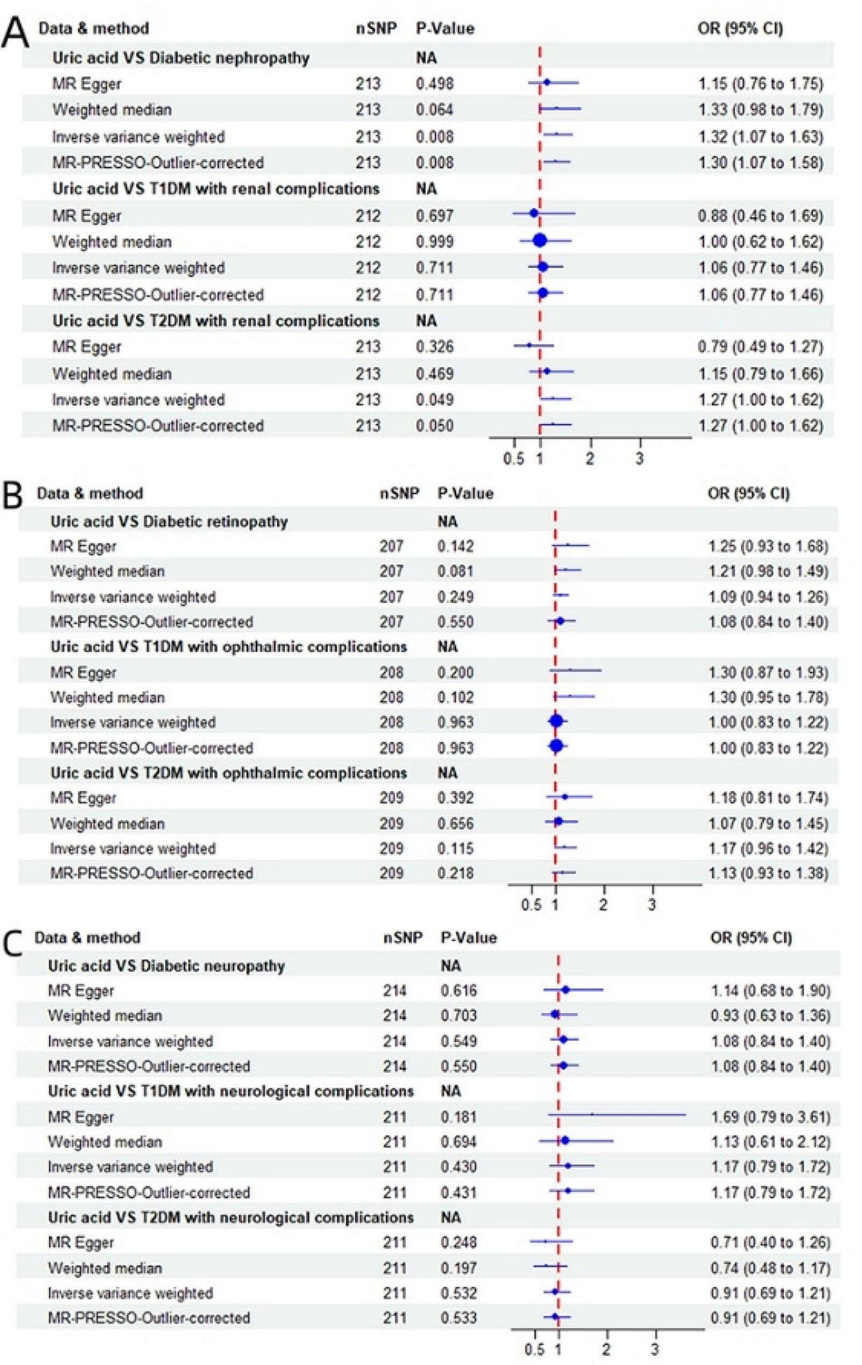
Forest plot of the association between SUA and diabetic microvascular complications. (**A**) Diabetic nephropathy, (**B**) diabetic retinopathy, and (**C**) diabetic neuropathy. The dot and bar indicate the causal estimate and 95%(CI) of the association between increasing SUA and diabetic microvascular complications, respectively

There was a causal relationship between genetically elevated SUA and diabetic nephropathy [OR = 1.32, 95%(CI) = 1.07–1.63, *p* = 0.008] according to the IVW model. The corrected MR-PRESSO model results [OR = 1.30, 95%(CI) = 1.07–1.58, *p* = 0.008] were consistent with the IVW results. The results of the MR‒Egger model [OR = 1.15, 95%(CI) = 0.76–1.75, *p* = 0.498] and WM model [OR = 1.33, 95%(CI) = 0.98–1.79, *p* = 0.064] were inconsistent with those of the IVW model. We also performed MR analysis on secondary outcomes of diabetic nephropathy [type 1 diabetes mellitus (T1DM) with renal complications and type 2 diabetes mellitus (T2DM) with renal complications]. There was no significant causal relationship between SUA and renal complications in any of the four models: the IVW model [OR = 1.06, 95%(CI) = 0.77–1.46, *p* = 0.711], the MR-PRESSO model [OR = 1.06, 95%(CI) = 0.77–1.46, *p* = 0.711], the MR‒Egger model [OR = 0.88, 95%(CI) = 0.46–1.69, *p* = 0.697], or the WM model [OR = 1.00, 95%(CI) = 0.62–1.62, *p* = 0.999]. However, we obtained the opposite results: the IVW model [OR = 1.27, 95%(CI) = 1.00-1.62, *p* = 0.049] and MR-PRESSO model [OR = 1.27, 95%(CI) = 1.00-1.62, *p* = 0.050] indicated a significant causal relationship between SUA and renal complications in patients with T2DM. The sensitivity analysis showed that no SNP had a significant effect on the causal relationship estimates in the “leave-one-out” analysis (Supplementary Fig. 1). The MR‒Egger intercept test in the pleiotropy analysis indicated that the IVs did not have any directional pleiotropy (Table 1). MR analysis found a significant causal relationship between SUA and both DN and T2DM with renal complications but no significant causal relationship between T1DM and renal complications (Fig. 3A).

**Table 1:** Pleiotropic and heterogeneous trials of causality between serum uric acid levels and the risk of diabetic microvascular complications

Exprosure	Outcome	Pleiotropy		Heterogeneity	
		MR‒Egger intercept test		Cochran’s Q test	
		Intercept	p value	Cochran’s Q	p value
Uric acid	Diabetic nephropathy	0.003	0.448	246.408	0.035
	T1DM with renal complications	0.004	0.516	260.005	0.012
	T2DM with renal complications	0.012	0.016	255.764	0.019
	Diabetic retinopathy	-0.002	0.437	327.399	1.88E-07
	T1DM with ophthalmic complications	-0.004	0.328	292.442	8.43E-05
	T2DM with ophthalmic complications	-0.001	0.903	262.897	0.007
	Diabetic neuropathy	-0.000	0.944	306.230	2.41E-05
	T1DM with neurological complications	-0.007	0.370	267.731	0.004
	T2DM with neurological complications	0.008	0.220	263.213	0.007

### Causal effect of SUA on diabetic retinopathy

No significant causal relationship existed between SUA and diabetic retinopathy according to the IVW [OR = 1.09, 95%(CI) = 0.94–1.26, *p* = 0.249], MR‒Egger [OR = 1.25, 95%(CI) = 0.93–1.68, *p* = 0.142], or WM models [OR = 1.21, 95%(CI) = 0.98–1.49, *p* = 0.081] of MR. The same conclusion was obtained from the corrected MR-PRESSO model [OR = 1.08, 95%(CI) = 0.84–1.40, *p* = 0.550]. MR analysis for secondary outcomes of diabetic retinopathy (T1DM with ophthalmic complications, T2DM with ophthalmic complications) was also performed. The results of the IVW model [OR = 1.00, 95%(CI) = 0.83–1.22, *p* = 0.963], MR-PRESSO model [OR = 1.00, 95%(CI) = 0.83–1.22, *p* = 0.963], MR‒Egger model [OR = 1.30, 95%(CI) = 0.87–1.93, *p* = 0.200], and WM model [OR = 1.30, 95%(CI) = 0.95–1.78, *p* = 0.102] showed no significant causal relationships between SUA and T1DM with ophthalmic complications. There was also no significant causal relationship between SUA and T2DM with ophthalmic complications according to the results of the four MR models, i.e., IVW [OR = 1.17, 95%(CI) = 0.96–1.42, *p* = 0.115], MR‒Egger [OR = 1.18, 95%(CI) = 0.81–1.74, *p* = 0.392], WM [OR = 1.07, 95%(CI) = 0.79–1.45, *p* = 0.665] and MR-PRESSO [OR = 1.13, 95%(CI) = 0.93–1.38, *p* = 0.218]. Similarly, the MR‒Egger intercept tests did not suggest any directional pleiotropy in IVs (Table 1). The details are listed in Supplementary Fig. 1. The results of MR analysis indicated that there was no significant causal relationship between SUA and diabetic retinopathy (Fig. 3B).

### Causal effect of SUA on diabetic neuropathy

The results of the IVW model [OR = 1.08, 95%(CI) = 0.84–1.40, *p* = 0.549], MR‒Egger [OR = 1.14, 95%(CI) = 0.68–1.90, *p* = 0.616], and WM models [OR = 0.93, 95%(CI) = 0.63–1.36, *p* = 0.703] showed no significant causal relationships between SUA and diabetic neuropathy. The same conclusion was obtained from the corrected MR-PRESSO model [OR = 1.08, 95%(CI) = 0.84–1.40, *p* = 0.550].

Moreover, we performed MR analysis on secondary outcomes of diabetic neuropathy (T1DM with neurological complications, T2DM with neurological complications) and showed that there was no significant causal relationship between SUA and either T1DM with neurological complications or T2DM with neurological complications. We used four models, namely, IVW [OR = 1.17, 95%(CI) = 0.79–1.72, *p* = 0.430], MR-PRESSO [OR = 1.17, 95%(CI) = 0.79–1.72, *p* = 0.431], MR‒Egger [OR = 1.69, 95%(CI) = 0.79–3.61, *p* = 0.181] and WM [OR = 1.13, 95%(CI) = 0.61–2.12, *p* = 0.694], to analyze the causal relationships between SUA and neither T1DM had neurological complications. Similarly, the relationships between SUA and T2DM with neurological complications were analyzed using the IVW model [OR = 0.91, 95%(CI) = 0.69–1.21, *p* = 0.532], MR‒Egger model [OR = 0.71, 95%(CI) = 0.40–1.26, *p* = 0.248], WM model [OR 0.74, 95%(CI) = 0.48–1.17, *p* = 0.197] and MR-PRESSO model [OR = 0.91, 95%(CI) = 0.69–1.21, *p* = 0.533]. Likewise, the MR‒Egger intercept tests suggested that there was no pleiotropy in the IVs (Table 1, Supplementary Fig. 1). The results of MR analysis indicated that there was no significant causal relationship between SUA and diabetic neuropathy (Fig. 3C).

## Discussion

The aim of this study was to investigate the causal relationship between an increase in SUA and diabetic microvascular complications using a two-sample MR approach. To the best of our knowledge, this is the first application of MR methods to comprehensively explore the association between SUA and the risk of diabetic microvascular complications. Our findings suggest a causal relationship between genetically predicted increased SUA and both diabetic nephropathy and T2DM with renal complications. However, there is no significant causal relationship between genetically predicted increased SUA and increased risk of diabetic retinopathy and diabetic neuropathy.

Diabetic nephropathy is a long-term microvascular complication of DM and the major cause of end-stage renal disease. SUA is associated with the occurrence and progression of diabetic nephropathy, and it is an independent risk factor for early kidney disease. A cross-sectional study of 20,464 adults with T1DM showed that each 1 mg/dl increase in SUA was associated with a 56% increase in DKD incidence and a 30% increase in albumin excretion [14]. SUA was significantly and positively correlated with both microalbuminuria and the risk of renal disease in patients with T2DM [15]. The results of our MR study suggested a causal relationship between genetically predicted increased SUA and diabetic nephropathy or between T2DM and renal complications, which supported the conclusions reached in previous observational studies. In another Italian study involving 1,449 T2DM patients with normal renal function, the cumulative incidence of new CKD was greater in patients with hyperuricemia (29.5%) than in those without hyperuricemia (11.4%, *p* = 0.001) after 5 years of follow-up [16]. These studies all assessed the relationship between baseline SUA concentrations and the progression of renal impairment while reducing some of the known confounders affecting the results [17, 18]. However, the SUA concentration was not causally linked to renal complications in T1DM patients in this study. A recent FinnDiane study in which SUA was measured in 3895 patients with T1DM used an MR approach to examine the causal relationship between SUA and DN; this study used 23 SNPs with good inferential quality as IVs. The results showed no causal relationship between SUA concentration and diabetic nephropathy in patients with T1DM [19]. The finding that reducing SUA levels are associated with a decreased risk of poor renal prognosis in diabetes patients in observational studies may be because the progression of diabetic nephropathy is the result of multiple factors working together, with SUA being only a secondary factor. A significant proportion of diabetes patients have kidney disease, not all of which is caused by diabetic nephropathy but rather by other factors common in the general population (such as age, obesity, and hypertension), leading to renal impairment. Most T1DM patients exhibit histological changes typical of diabetic nephropathy. In young patients with uncomplicated T1DM, SUA levels are usually lower due to elevated blood glucose. This may explain why no causal association between uric acid and renal complications in patients with T1DM was observed in this study. Future research will provide important new data to clarify the true role of uric acid in diabetic nephropathy.

A meta-analysis of 35 studies from around the world showed that the overall prevalence of diabetic retinopathy among diabetic patients was 34.6%, and the overall prevalence of vision-threatening diabetic retinopathy (VTDR) was 10.2%. VTDR has become one of the major causes of acquired blindness in working-age adults worldwide [20]. In a study of 385 patients with T2DM, high SUA was a risk factor for DR (OR = 1.264, 95%(CI) = 1.08–1.473, *p* = 0.003) [21], and a similar finding was reported in Japan [22]. However, a cross-sectional study in China found that elevated SUA was an independent protective factor for DR (OR = 0.997, 95%(CI) = 0.995–0.999, *p* = 0.018) [23]. A meta-analysis that included 4340 diabetic retinopathy patients and 8595 placebo patients revealed a linear dose‒response correlation of elevation in diabetic retinopathy patients with different SUAs, which progressively increased from nonsignificant to significant. The results of the study suggest that proliferative diabetic retinopathy (PDR) participants had a significantly greater SUA than diabetic control participants, whereas there was no significant difference in non-proliferative diabetic retinopathy (NPDR) patients (WMD = 22.50, 95%(CI)=-6.07-51.08, *p* = 0.120, I2 = 97%, *P* < 0.001). The 21 studies included in this meta-analysis had significant heterogeneity and did not control for the use of anti-hyperuricemia medications, which may have influenced the final conclusions [24]. The results of our MR study suggested that genetically predicted increased SUA levels were not significantly causally related to the risk of diabetic retinopathy.

Diabetic neuropathy is one of the most common chronic complications of DM, and the common types of diabetic neuropathy are distal symmetric polyneuropathy (DSPN) and autonomic neuropathy. Previous studies have shown an association between SUA and an increase in the risk of diabetic neuropathy. A recent study included 230 T2DM patients to explore the relationship between SUA levels and DPN in patients with T2DM and divided them into a DPN group and a non-DPN group according to whether DPN was diagnosed [5]. The average SUA level in the DPN group was significantly greater than that in the control group [(6.72 ± 1.75) vs. (4.57 ± 1.49) mg/dl]. Logistic regression analysis suggested that with increasing SUA, the risk of DPN increased 2.2-fold. Another study showed that SUA ≥ 434 µmol/L was significantly associated with an increase in DPN compared to SUA < 262 µmol/L (OR = 1.54, 95%(CI) = 1.02–2.32) [21]. Hyperuricemia can contribute to the progression of diabetic neuropathy by causing vascular dysfunction, thrombosis, and the inhibition of NO release. According to the results of a meta-analysis that included 6134 patients, hyperuricemia was shown to be independently associated with an elevated risk of diabetic neuropathy in patients with T2DM [25]. However, a cross-sectional study revealed no association between hyperuricemia and diabetic polyneuropathy. In addition, in experimental animal studies, SUA was shown to have neuroprotective effects on dopaminergic neurons in Parkinson’s disease mice by modulating neuroinflammation and oxidative stress [26]. Due to the strong hydrophilic antioxidant effect of SUA, to some extent, this potential neuroprotective property may play an important role in neurodegenerative diseases [27]. Our MR findings suggested that genetically predicted increased SUA was not significantly causally related to the risk of diabetic neuropathy.

Since there is a lack of large-scale randomized controlled trials to draw definitive conclusions, we included a fairly large genetic homogeneity and well-characterized patient population in our MR analysis, and all SNPs identified as IVs were from European populations. This approach reduced the possibility of population stratification bias and enhanced the validity of the two-sample MR hypothesis. In addition, the strong correlation IVs (F statistic significantly more than 10) that we used in this study can mitigate the potential bias of sample overlap. Moreover, the selected SNPs were associated with SUA concentration but not with the outcome factor (diabetic microvascular complications). None of our sensitivity analyses suggested the presence of significant pleiotropy in which a single gene affects multiple traits. However, there are some limitations in our study. First, our exposure factors were obtained from the UKB queue, so this study lacked other GWAS summary statistics for positive control analysis. Second, since only summary-level GWAS statistics were available, there were no further subgroup analyses. Finally, our study is descriptive and therefore provides no insight into the underlying mechanisms of the progression of diabetic microvascular complications.

## Conclusions

In conclusion, our MR analysis suggested a causal relationship between genetically predicted increases in SUA and diabetic microvascular complications. There was a significant causal relationship between SUA and diabetic nephropathy but not between SUA and diabetic retinopathy or diabetic neuropathy. This study contributes to providing evidence on the causal relationship between SUA and diabetic microvascular complications.

### Electronic supplementary material

Below is the link to the electronic supplementary material.

Supplementary Material 1

Supplementary Material 2

## Data Availability

The datasets used and/or analyzed in the current study are available from the corresponding author upon reasonable request.
