# Supplementary material for: Causality between serum uric acid and diabetic microvascular complications - a mendelian randomization study
Source: Diabetol Metab Syndr. 2024 Jun 18;16:134. doi: 10.1186/s13098-024-01377-x (PMC11186091; doi:10.1186/s13098-024-01377-x)
Supplement: Supplementary file 2 — Supplementary Material 2 [file 13098_2024_1377_MOESM2_ESM.docx]

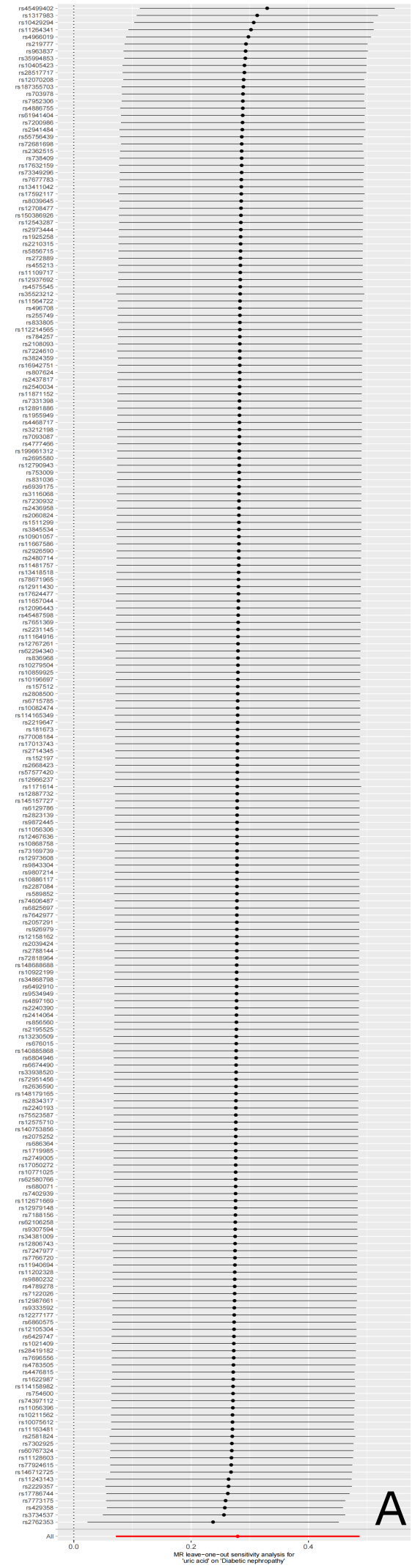

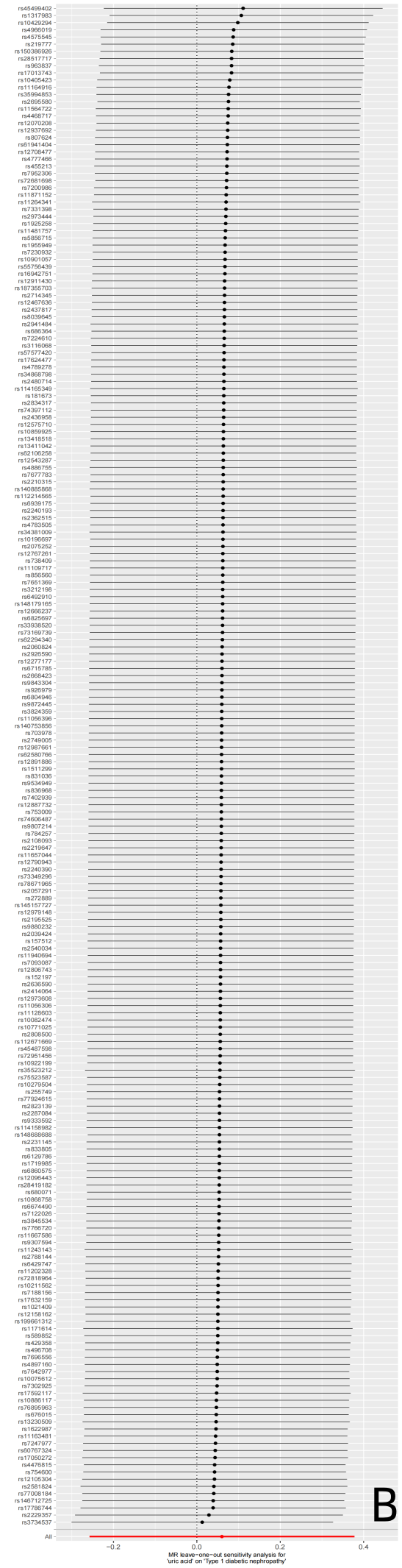


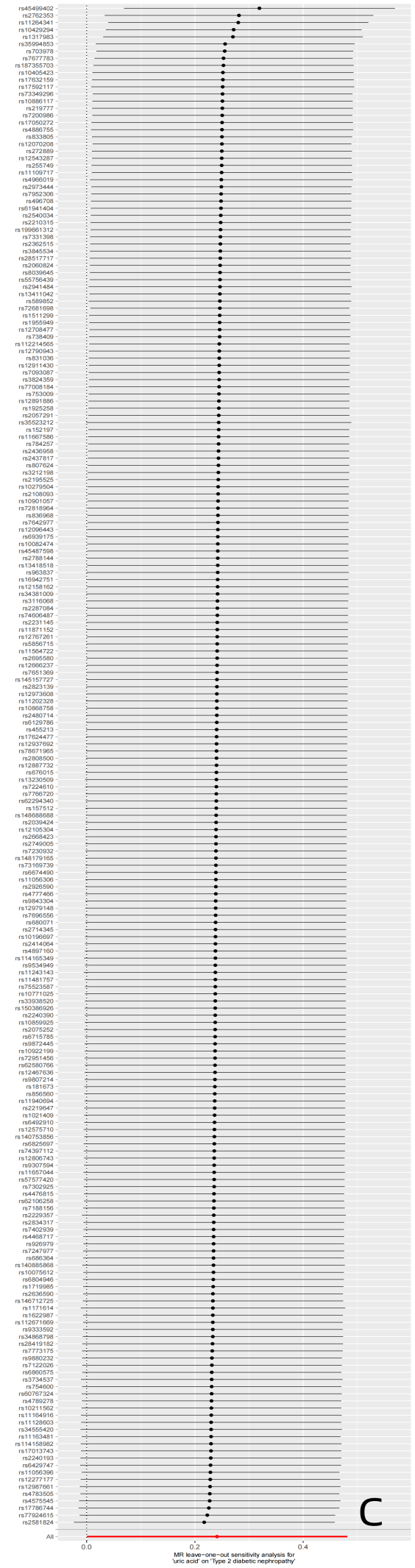

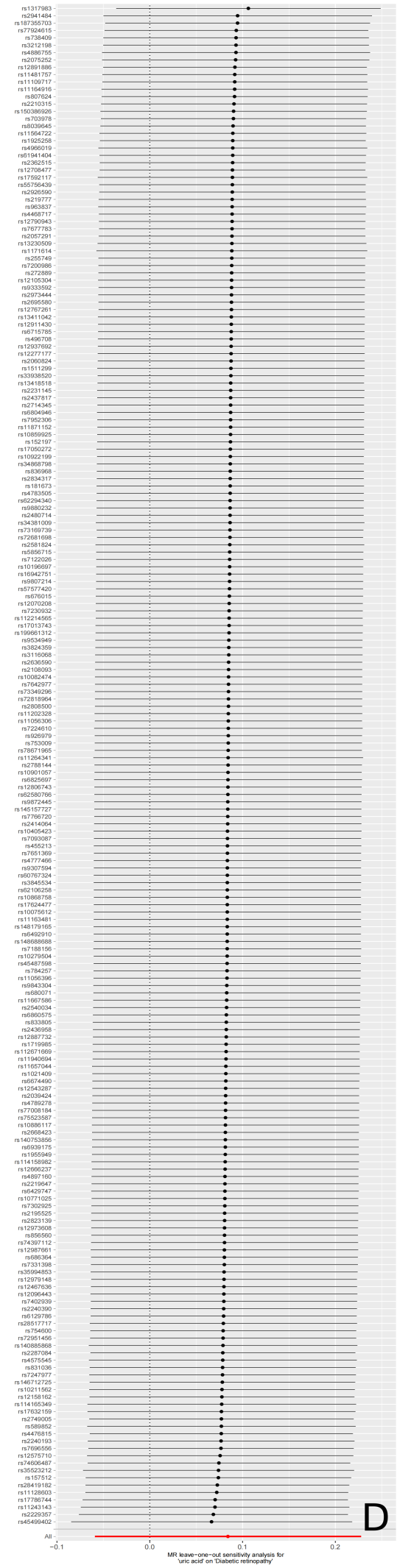


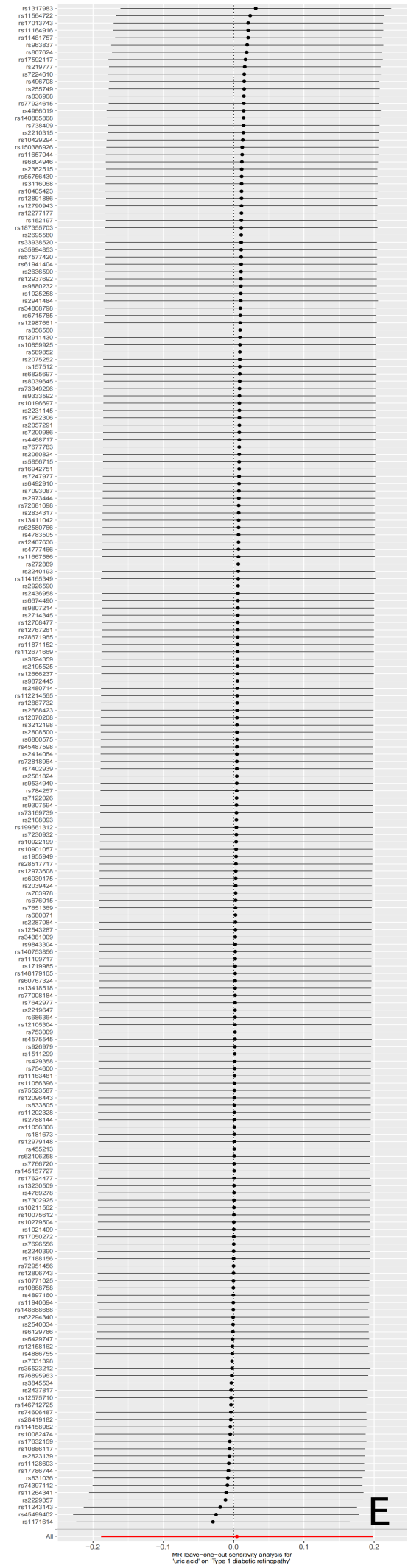

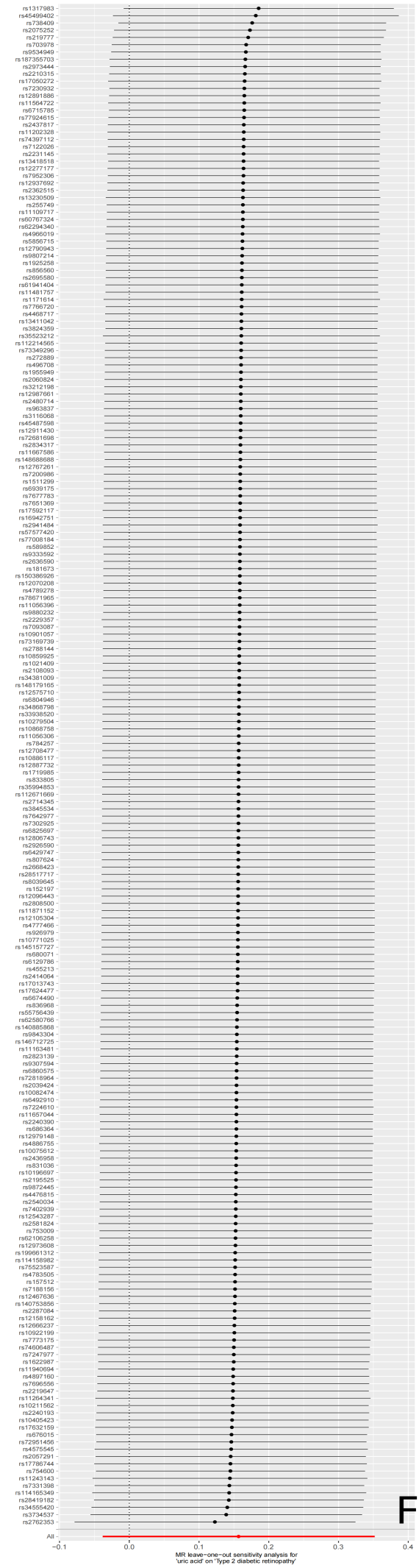


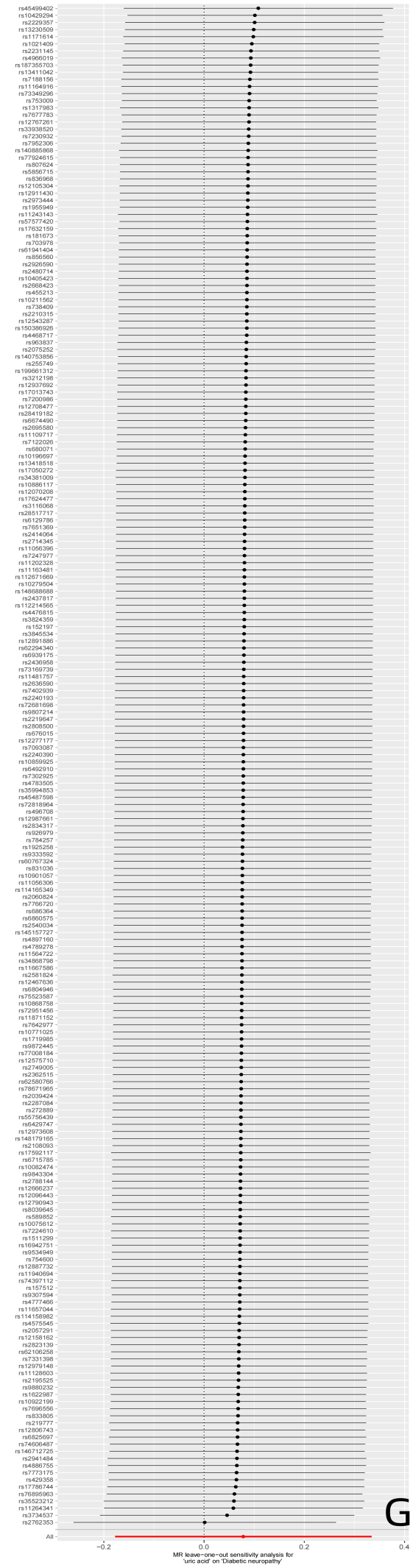

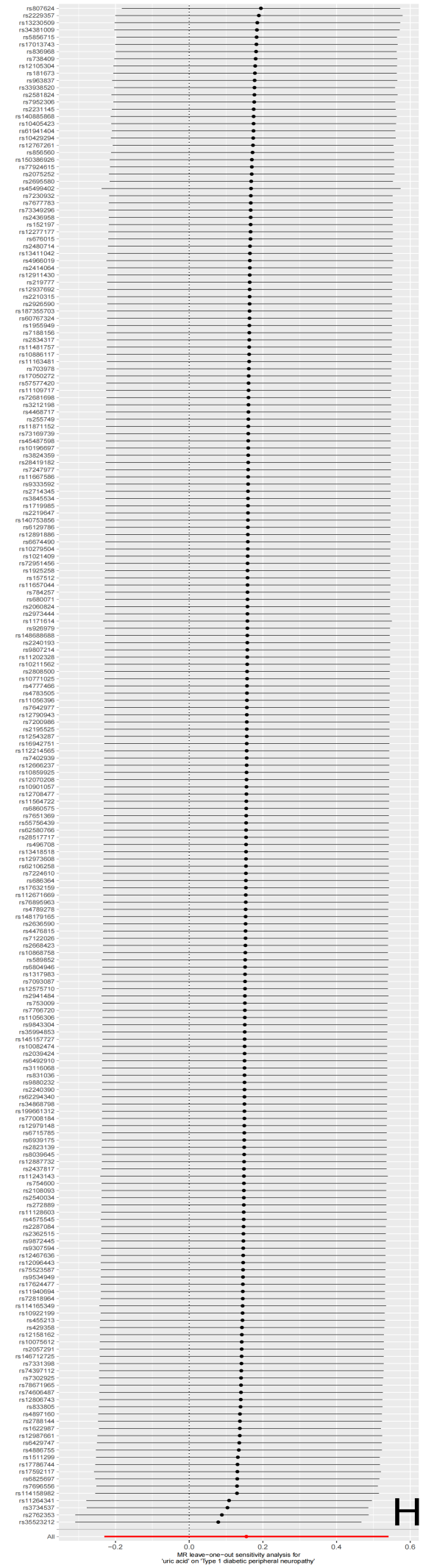


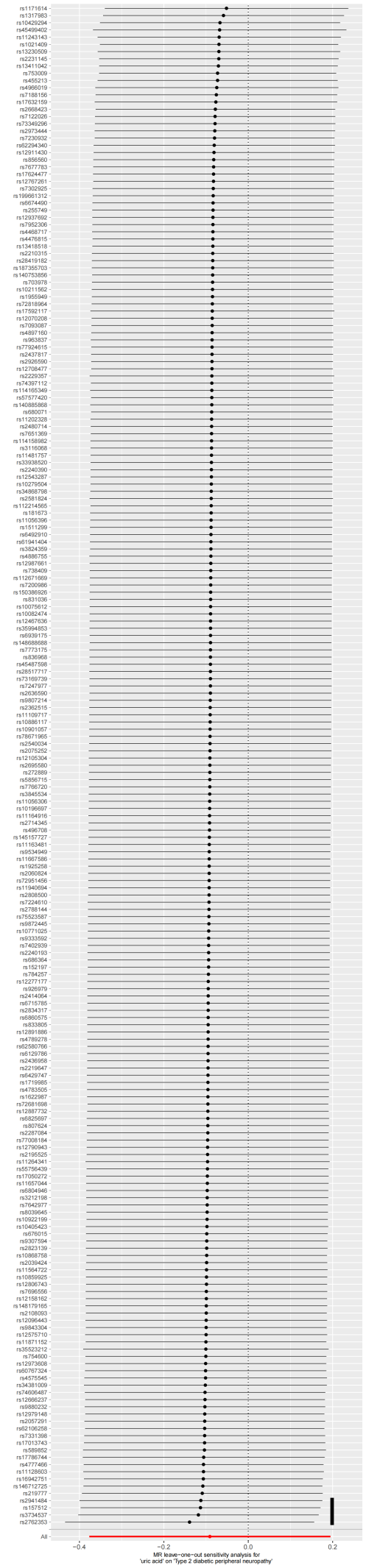


Supplementary Figure 1. The plots of “leave-one-out” analysis method to show the influence of individual SNP on the causal effect of genetically predicted SUA on diabetic microvascular complications.（A）Diabetic nephropathy;（B）T1DM with renal complications;（C）T2DM with renal complications;（D）Diabetic retinopathy（E）T1DM with ophthalmic complications;（F）T2DM with ophthalmic complications;（G）Diabetic neuropathy;（H）T1DM with neurological complications;（I）T2DM with neurological complications
